# Thermoresponsive 2-hydroxy-3-isopropoxypropyl hydroxyethyl cellulose with tunable LCST for drug delivery†

**DOI:** 10.1039/c8ra09075k

**Published:** 2019-01-16

**Authors:** Ye Tian, Ying Liu, Benzhi Ju, Xiaozhong Ren, Mingyun Dai

**Affiliations:** Aquacultural Engingeering R&D Center, Dalian Ocean University Dalian 116023 China tianye@dlou.edu.cn +86 411 84763255; State Key Laboratory of Fine Chemicals, Dalian University of Technology Dalian 116024 China jubenzhi@dlut.edu.cn +86 411 84986269; Key Laboratory of Mariculture & Stock Enhancement in North China's Sea, Dalian Ocean University Dalian 116023 China

## Abstract

Thermoresponsive polymer 2-hydroxy-3-isopropoxypropyl hydroxyethyl celluloses (HIPECs) were successfully synthesized, characterized, and applied for thermoresponsive drug delivery. The lower critical solution temperature (LCST) of HIPEC could be easily tuned from 21.1 to 56.1 °C as the molar substitution (MS) increased from 1.21 to 2.88. Dynamic light scattering and transmission electron microscopy experiments revealed that HIPEC can self-assemble into nano-sized aggregates, and their size could be changed by variation in temperature. Additionally, the critical aggregation concentration (CAC) ranged from 0.101 to 0.805 g L^−1^ by changing MS of HIPEC. *In vitro* drug delivery studies indicated that the amphotericin B (AmpB) release rate was much faster at temperatures above LCST; approximately 95% of the drug was released from aggregates in 40 h. MTT assays were conducted to evaluate the cytotoxicity of HIPEC, and the observation of the Hoechst 33342 living cell stain using confocal laser scanning microscopy confirmed the high cell viability as HIPECs were used.

## Abstract 2

Thermoresponsive polymer 2-hydroxy-3-isopropoxypropyl hydroxyethyl celluloses (HIPECs) were successfully synthesized, characterized, and applied for thermoresponsive drug delivery.

## Graphical abstract

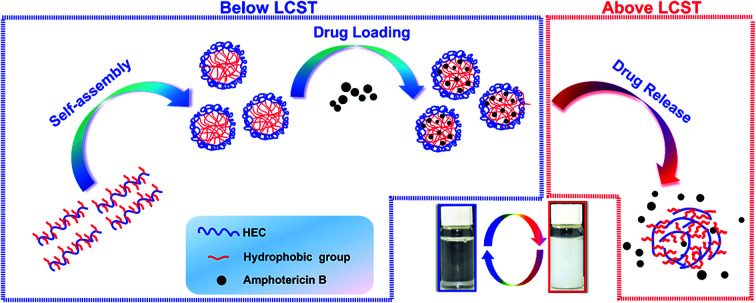

## 1. Introduction

Stimuli-responsive polymers have received much attention in recent decades. The development of thermoresponsive polymers has especially experienced significant advances because of their technical feasibility and practical advantages.^1,2^ Poly(*N*-isopropylacrylamide) (PNIPAM) and its derivatives exhibit an abrupt coil-to-globule transition in water at the lower critical solution temperature (LCST) of 32 °C,^3^ and they have been widely developed into biomaterials, especially polymeric aggregates, for the controlled delivery of various drugs. The reasons for this biomedical popularity are that LCST of PNIPAM is not only close to body temperature, but also not apparently affected by environmental conditions.^4,5^ Generally speaking, thermoresponsive polymers with LCSTs in the range of 20–40 °C, which are affected slightly by different biological environments, are promising candidates for biomedical applications.^6–11^

Thermoresponsive polymers have attracted considerable interest in many application fields; the most promising application is drug delivery. For this purpose, amphiphilic thermoresponsive polymers that can self-assemble into aggregates are particularly attractive. The aggregates or micelles providing a potential carrier for hydrophobic drugs have many advantages including toxicity reduction, drug bioavailability improvement, and drug hydrolysis protection. The key point of efficient drug delivery system design is that the release of drugs should be controlled release in time and space. The thermoresponsive drug delivery systems, if properly designed, can realize controlled release in very mild conditions even in the physiological temperature range.^6,12–14^ Typical studies have focused on block copolymers containing poly(*N*-isopropylacrylamide) and poly(alkylene oxide) functional units. These thermoresponsive copolymers can self-assemble into stable spherical micelles or cylindrical aggregates in the appropriate temperature range. When the temperature reaches LCST, micelle aggregates are formed due to the dehydration and collapse of the outer shell chains, and the drugs encapsulated in aggregates can be released over a much longer period of time. Recently, cellulose-based thermoresponsive materials that can self-assemble have been shown to have tremendous potential for applications as drug carriers. These kinds of cellulose-based materials can control the drug release. The concept of using cellulose materials for drug delivery shows great promise.

The value of LCST is the crucial parameter for drug delivery systems based on thermoresponsive polymers, and LCST needs to be changed along with different living organisms in biomedical applications. Thus, it is essential to develop a convenient and efficient method to tune the LCST of thermoresponsive polymers.^15–17^ It is essential to note that LCST is affected by the ratio of hydrophobic monomers to hydrophilic ones in thermoresponsive polymers. Therefore, LCST can be tuned by the ratio variation of these groups. For example, LCSTs of poly[oligo(ethylene glycol)methyl vinyl ether] and poly(*N*,*N*-dialkyl acrylamide) can be tuned by changing the lengths of the hydrophilic oligo(ethylene glycol) groups or the types of hydrophobic alkyls.^18,19^

PNIPAM, poly(alkylene oxide) and their derivatives are perhaps the most extensively researched among various thermoresponsive materials applied in drug delivery systems.^19,20^ Drug delivery is expected to be used *in vivo*; the concern for the cytotoxicity of thermoresponsive materials is unavoidable. Unfortunately, PNIPAM, poly(alkylene oxide) and their derivatives have low biocompatibility, which limits their potential application as drug carriers. In contrast, cellulose has natural compounds, which have many advantageous properties including nontoxicity, abundance, and high biocompatibility.^21–24^ Grafting thermoresponsive polymers onto the main chains of cellulose and its derivatives is an efficient method, which can improve the biocompatibility of thermoresponsive materials.^25,26^ For example, the LCST values of cellulose-based thermoresponsive polymers, *i.e.*, nanocrystals-*g*-poly(poly(ethylene glycol) methylacrylate)s, cellulose-*g*-poly(*N*,*N*-diethylacrylamide), and hydroxypropyl cellulose-g-PNIPAM are 34–66, 18–26, and 28–42 °C, respectively.^27–29^ These grafted copolymers combine the virtues of the thermal responsiveness of synthetic polymers and the biocompatibility of cellulose and its derivatives. In recent years, increasing number of publications have demonstrated that self-assembly as a feasible and effective strategy to prepare cellulose-based thermoresponsive materials has been deeply studied. These materials, which are prepared *via* the grafting copolymerization method, can be suitable candidates as drug carriers. It is worth noting that LCSTs of these grafting copolymers are nearly similar to LCSTs of the corresponding grafted thermoresponsive polymer segments; this indicates that it is difficult to tune the LCSTs of these thermoresponsive copolymers, resulting in the limitation of their practical use as drug delivery carriers.

On the basis of these considerations, we used hydrophobic isopropyl glycidyl ether (IPGE) to graft onto hydrophilic hydroxyethyl cellulose (HEC), synthesizing thermoresponsive and biocompatible 2-hydroxy-3-isopropoxypropyl hydroxyethyl celluloses (HIPECs) with sharp and easily tunable LCST. As expected, amphiphilic, thermoresponsive HIPECs could form aggregates and encapsulate guest molecules in aqueous solutions. Fluorescence spectroscopy was used to determine CAC of HIPECs. In this study, amphotericin B was selected as the model drug to evaluate the potential of HIPEC aggregates as thermoresponsive drug carriers. The release behaviors of amphotericin B below and above LCST were investigated. In addition, the cytotoxicity of HIPEC was evaluated against normal human hepatocytes (HL-7702 cells) using MTT assays and confocal laser scanning microscopy experiments. We suggested that the studied HIPECs may have great potential to be used as thermoresponsive drug carriers *in vivo*.

## 2. Experimental

### 2.1. Materials

Hydroxyethyl cellulose was obtained from Sigma-Aldrich (USA, MSOH = 2.5, MW = 3.64 × 10^5^ g mol^−1^, PDI = 2.15). Isopropyl glycidyl ether was obtained from Tokyo Chemical Industry Co., Ltd. Amphotericin B was obtained from Sigma-Aldrich. All other reagents were used as received without further purification.

### 2.2. Methods

In a 100 mL three-necked flask, 2.0 g HEC (7.3 mmol of anhydroglucose units AGU), 10 mL deionized water, and 1.0 g NaOH aqueous solution (40 wt%) were added; the mixture was placed in a 60 °C water bath under stirring for 1 h. A predetermined amount of isopropyl glycidyl ether (IPGE) was added dropwise to the flask. The reaction was continued for 5 h, and the temperature was kept at 90 °C. At the end of the reaction, the mixture was cooled to room temperature and neutralized to pH 7.0 with 6 M HCl. HIPECs were purified *via* dialysis in deionized water for four days, followed by concentration by a rotary evaporator and then drying by lyophilization.

### 2.3. Characterization

^1^H-NMR, ^13^C-NMR and 2D HSQC NMR spectra were executed on a Varian INOVA 500 spectrometer at room temperature. HIPECs (30 mg) were dissolved in 1 mL DMSO-*d*_6_.

The LCST values of HIPECs were measured by Mettler Toledo T90 with a thermo-controlled LAUDA RP200 (Germany). LCST of HIPECs in aqueous solutions (5 g L^−1^) is defined as the temperature where 50% of the absorbance transition occurs.

The critical aggregation concentrations of HIPECs were measured with a fluorescence spectrophotometer (Hitachi F 7000, Japan) using pyrene as the hydrophobic probe. The pyrene concentration used was 1.6 × 10^−6^ mol L^−1^. Pyrene was excited at 373 nm, and the excitation spectra were scanned from 300 to 360 nm. The measurements were carried out at 25 °C. Gel permeation chromatography (GPC, Agilent Technologies 1200 series, USA) was carried out to determine the molecular weights and molecular weight distributions (more details about the GPC experiment are presented in the ESI†). Dynamic light scattering (DLS Malvern Nano-ZS90, Britain) was performed to determine the average diameters of the aggregates of HIPECs. The processes of operation were similar to previously reported procedures.^30^

The morphologies of the HIPEC aggregates were observed using transmission electron microscopy (FEI TF30) at an accelerating voltage of 80 kV. The aggregate solutions were prepared below the LCST or heated up to the LCST. Then, a small drop from the aggregate solutions was deposited onto the carbon-coated copper TEM grid at room temperature. Excess HIPEC solution was removed using a filter paper and dried in air.^31^

### 2.4. Drug release behaviors *in vitro*

Amphotericin B (AmpB, 5 mg) and HIPEC-3 (30 mg) were dissolved in 2 mL of DMSO; subsequently, the solution was added into 20 mL of deionized water and stirred for 50 min at 10 °C. After this, the solution was dialyzed against water for 48 h by a 3000 Da molecular weight dialysis tube, and the water was changed every 6 h at 10 °C. In order to remove excess drug that was not dialyzed from the dialysis tube, the dialyzed solution was filtered with a 0.45 mm syringe. The drug content encapsulated in the aggregates was determined by UV spectroscopy, and the loading efficiency was calculated using eqn (1). The solution was dropped into a dialysis tube again and placed in 20 mL of deionized water in a super thermostatic water bath at 35 or 38 °C. At preset time intervals, the release medium was recycled for analysis and renewed with new deionized water. The drug concentration released from HIPEC-3 aggregates was measured by UV spectroscopy at 388 nm. The cumulative release was calculated by using eqn (2) as follows:1
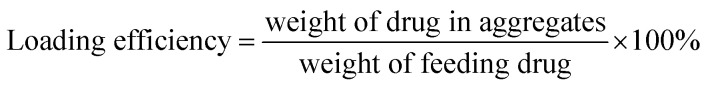
2
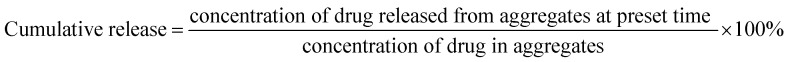


### 2.5. Cytotoxicity

Normal human hepatocytes (HL-7702 cells) were purchased from the Institute of Basic Medical Sciences (IBMS) of the Chinese Academy of Medical Sciences and cultured in RPMI-1640 medium (Hyclone) supplemented with 10% fetal bovine serum (Invitrogen) at 37 °C under a mixture of 5% CO_2_ and 95% air atmosphere.

Measurement of cell viability was evaluated by reducing MTT (3-(4,5-dimethylthiazol-2-yl)-2,5-diphenyltetrazolium bromide) to formazan crystals using mitochondrial dehydrogenases in living cells (Mosmann, 1983). Then, 100 μL medium containing HL-7702 cells and 10% fetal bovine serum (FBS) was seeded in the 96-well culture cluster (Costar) at a density of 1 × 10^5^ cells per mL per well. After cell attachment for 24 h, the cluster was then washed with PBS 100 μL per well (0.01 M, pH = 7.4). The cells were then cultured in a medium containing 10% FBS with 8, 16, 32, 64, 125, 250, 500, 1000 μM of HIPECs for 12 h. Cells in the culture medium without HIPECs were used as the control group. Six replicate wells were used for the control and experimental groups. Next, 10 μL of MTT (5 mg mL^−1^) prepared in PBS was added to each well, and the culture cluster was incubated at 37 °C in a 5% CO_2_ humidified incubator. After 4 h, the medium was carefully removed, and the purple crystals were dissolved in 200 μL DMSO per well. The optical density of every well was detected in a microplate reader (Thermo Fisher Scientific, USA) at 490 nm and 570 nm wavelengths. Cell viability of the experimental groups was expressed as a percentage relative to that of the control group, and it was calculated using the following equation:3
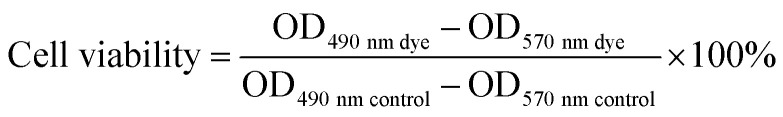


HL-7702 cells were seeded in confocal 24-well flat-bottomed plates with 10^5^ cells per dish and cultured for 24 h in RPMI-1640 medium (Hyclone) supplemented with 10% fetal bovine serum (Invitrogen) at 37 °C under a mixture of 5% CO_2_ and 95% air atmosphere. HIPEC-3 (5 g L^−1^) was added to each dish and cultured at 37 °C in the culture medium. After 24 h culture, Hoechst 33342 (2 μM, 25 min) and propidium iodide (PI, 3 μM, 15 min) were sequentially added to incubate the cells in the culture medium without FBS for different durations. To avoid the background fluorescence, the cells were washed by PBS (0.01 M, pH = 7.4) three times, and 1 mL of serum-free medium was finally added before imaging. Cells were imaged with a 60× oil-immersion objective lens in an inverted-type scanning confocal microscope (Olympus FV1000, USA). Hoechst 33342 was excited with a 405 nm laser, and the fluorescence emission was collected in the 421–501 nm wavelength range in channel 1 (blue color). PI was excited with a 559 nm laser, and the fluorescence emission was collected in the 577–657 nm wavelength range in channel 2 (red color).

## 3. Results and discussion

### 3.1. Synthesis and characterization of HIPECs

**Scheme 1:**
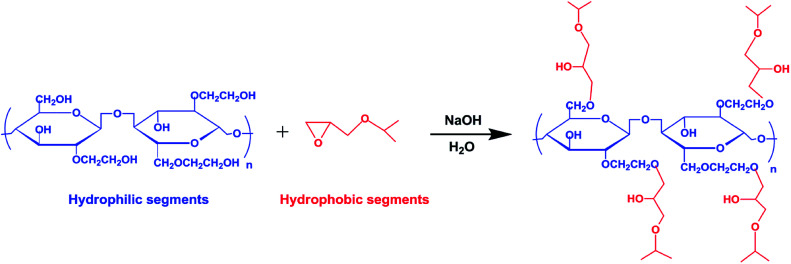
Synthesis pathway of thermoresponsive HIPECs.

2-Hydroxy-3-isopropoxypropyl hydroxyethyl celluloses were synthesized *via* an etherification reaction of hydroxyethyl cellulose with isopropyl glycidyl ether, as illustrated in Scheme 1. The characteristics and solution properties of HIPECs with different MS are shown in Table 1.

**Table 1:** Preparation and characterization of HIPECs

Code	IPGE^a^ : AGU^b^	MS^c^	LCST/°C	CAC^d^ at 20 °C (g L^−1^)	*M*_W_ e × 10^5^/(g mol^−1^)	PDI^e^	Loading efficiency (%)
HIPEC-1	2.5	1.21	56.1	0.805	2.99	6.51	7.1
HIPEC-2	3.0	1.51	48.6	0.689	2.36	7.70	8.9
HIPEC-3	3.5	2.01	37.3	0.355	2.56	3.18	11.9
HIPEC-4	4.0	2.36	30.2	0.234	2.26	6.93	13.7
HIPEC-5	4.5	2.88	21.1	0.101	2.45	7.72	15.4

a IPGE, isopropyl glycidyl ether.

b AGU, anhydroglucose unit.

c MS, molar substitution.

d CAC, critical aggregation concentration.

e Determined by GPC.

The structure of HIPECs was verified by ^1^H-NMR, ^13^C-NMR, and 2D HSQC NMR spectra. The ^1^H-NMR and ^13^C-NMR spectra of HIPEC-3 are shown in Fig. S1 and S2, respectively, of the ESI.† The peak at 1.09 ppm could be assigned to an isopropyl group (H13, H13′); the double peaks at 4.35–4.80 ppm were attributed to the anomeric proton (H1 and H1′) of HIPEC-3, indicating partial substitution at the hydroxyl in the 2-O position. The broader signal between 2.70 and 4.00 ppm corresponded to the protons of the anhydroglucose units and the protons of the O(CH_2_CH_2_O)_*n*_–CH_2_–CHOH–CH_2_–O–CH_2_– group. MS of HIPECs was calculated using the ratio of the integral of methyl in the isopropyl group to six times the integral of H1 in AGU by ^1^H-NMR. In ^13^C-NMR, the characteristic peak for a methyl group (C13 and C13′) was identified at 22.58. It is worth noting that a C1 and a C1′ signal occurred at around 102 ppm, also indicating partial substitution at hydroxyl of 2-O position. All of these results indicated successful etherification. The ^1^H peak and ^13^C assignment of HIPEC/DMSO-*d*_6_ were further confirmed with 2D HSQC NMR spectra (Fig. S3†), offering proof of one-bond correlation between protons and carbon.

### 3.2. Thermoresponsive behaviors of HIPECs

**Fig. 1:**
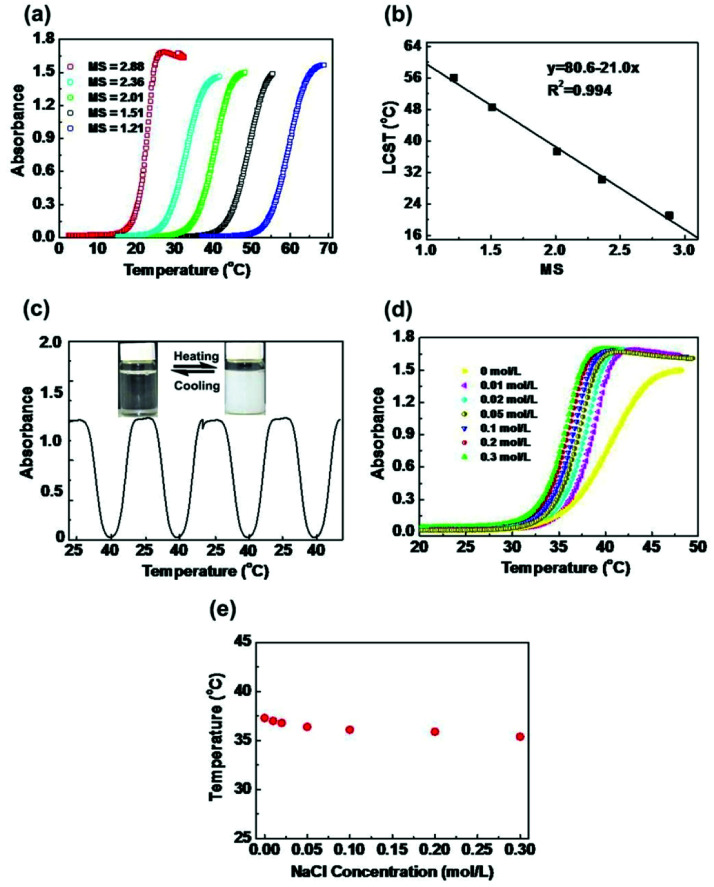
(a) Absorbance changes for HIPEC-1∼5 aqueous solutions (5 g L^−1^) with a heating rate of 1 °C min. (b) Effect of MS on LCST. (c) Reversible changes of optical absorbance against temperature fluctuation for HIPEC-3 aqueous solutions (5 g L^−1^) (inset: photographs of HIPEC-3 aqueous solution at 25 °C and 40 °C). (d) Absorbance variations of HIPEC-3 aqueous solutions (5 g L^−1^) with different NaCl concentrations during heating. (e) Effect of NaCl concentration on LCST.

The thermoresponsive behaviors of HIPECs in aqueous solutions were investigated at a concentration of 5 g L^−1^. As shown in Fig. 1(a), the absorbance increased in all HIPEC solutions with different MS values upon heating, indicating LCST transition. When the temperature was below the LCST, the hydrophilic groups (HEC skeleton groups) of HIPECs forming strong hydrogen bonds with water molecules contributed to HIPEC dissolution in water. As the temperature increased, hydrogen bonding between the hydrophilic groups and water weakened, while hydrophobic group (isopropyl groups) interactions strengthened among the side groups. When the temperature increased above LCST, the interactions between hydrophobic chains became dominant, resulting in an entropy-driven HIPEC chain collapse and phase separation.^4,32^ After comparing one absorbance curve with another, it appears that the HIPEC solution has higher MS and lower LCST in water. As illustrated in Fig. 1(b), LCST of HIPECs decreased linearly with increasing MS, and the increase in MS from 1.21 to 2.88 caused notable decrease in LCST from 21.1 to 56.1 °C, indicating that the LCST of HIPECs can be tuned by simply changing the MS of isopropyl groups. In our case, the LCST of HIPECs could be precisely adjusted according to application requirements. The aim of the present work is to use HIPECs as thermoresponsive carriers in drug delivery systems. Therefore, in this paper, 5 g L^−1^ of HIPEC-3 sample solution was chosen for further study because of the LCST of 37.3 °C, which is close to the human body temperature. Aqueous solutions of HIPECs could exhibit reversible phase separation behaviors. As shown in Fig. 1(c), the absorbance values of the HIPEC-3 solution during heating and cooling thermal cycles between 25.0 °C and 40.0 °C were approximately similar to each other in the multiple-cycle experiments.

The thermoresponsive polymers utilized in biomedical applications should be insensitive to environmental conditions; specifically, the LCST of polymers should not be apparently affected by slight variations in pH, ionic strength, or concentrations.^4^ The most important parameter of the environmental conditions for biomedical application is the influence of salts on the thermoresponsivity of materials. Thus, the influence of salt on the LCST of HIPECs was investigated (Fig. 1(d)). The LCST of HIPEC-3 decreased with increasing amounts of sodium chloride. The salt present in HIPEC-3 aqueous solution led to partial dehydration of the HIPEC molecular chain and consequent decrease in LCST. Nevertheless, the LCST only decreased by 2.8 °C when the NaCl concentration increased from 0 to 0.2 mol L^−1^ (Fig. 1(e)). This indicated that slight variation in salt concentration affected the LCST of HIPECs by only a few degrees.

### 3.3. Self-assembled aggregates of HIPECs

**Fig. 2:**
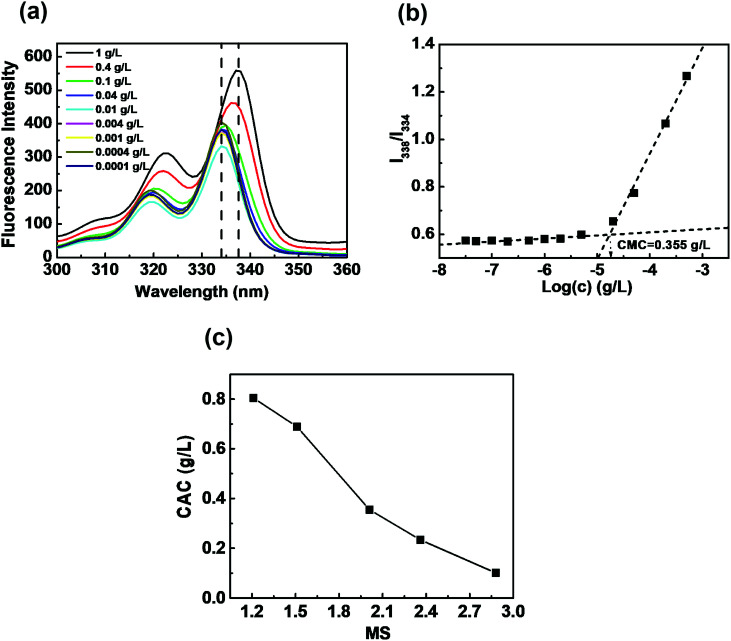
(a) Excitation spectra of pyrene (6 × 10^−7^ mol L^−1^) in water in the presence of increasing concentrations of HIPEC-3 (*λ*_em_ = 490 nm). Measurements were performed at 10 °C. (b) Plot of *I*_338_/*I*_334_ in the excitation spectra *versus* the concentration of HIPEC-3. (c) Effect of MS on CAC.

Amphiphilic HIPECs tend to form aggregates by self-assembly in aqueous solutions using the direct dissolution method. HIPEC aggregates could be used as potential carriers for controlled drug delivery. The critical aggregation concentration as an important parameter was adopted to evaluate the stability of aggregates. CACs could be determined by fluorescence spectroscopy with the aid of pyrene, which was used as a probe to investigate the aggregate-forming behavior of HIPECs. A sequence of the fluorescence excitation spectra of pyrene under different HIPEC-3 concentrations at room temperature (below the LCST) is illustrated in Fig. 2(a). As the HIPEC-3 concentration increased from 0.0001 to 1 g L^−1^, it was clear that the fluorescence intensity of pyrene increased, followed by a red shift in the excitation spectra from 334 to 338 nm. This demonstrated aggregate formation by self-assembled HIPEC-3 and pyrene encapsulated into hydrophobic domains of aggregates from the water media. The peak height-intensity ratio (*I*_1_/*I*_3_) values of pyrene fluorescence spectroscopy were plotted as a function of HIPEC-3 concentration (Fig. 2(b)), and CAC was measured to be approximately 0.355 g L^−1^. In addition, the CAC values of HIPECs could be tuned by changing the MS of isopropyl groups. Specifically, the CAC values of HIPECs decreased from 0.805 to 0.101 g L^−1^ with the increase in MS (Fig. 2(c) and Table 1). In other words, a higher isopropyl group content led to lower CAC. The enhancement in HIPEC hydrophobicity induced by MS increase facilitated the interactions among the hydrophobic chains of HIPECs, resulting in decrease in CAC.

**Fig. 3:**
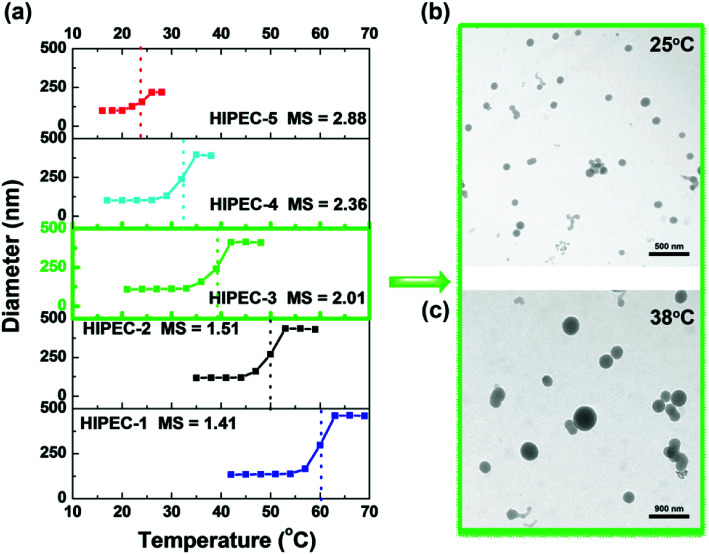
(a) Plots of the average hydrodynamic diameter of HIPECs with different MS *versus* temperature. TEM images (b) of HIPEC-3 aggregates observed at 25 °C and (c) large aggregates formed after heating the solution to 38 °C.

Further investigations into the thermoresponsivity of HIPEC aggregates were conducted by dynamic light scattering. For HIPEC-1∼5 aqueous solutions, the size of the aggregates remained constant below their LCST (Fig. 3(a)). When the temperatures of sample solutions were increased beyond their LCST, the association of aggregates occurred. For example, the aggregate diameter of HIPEC-3 significantly increased from 110 to 328 nm as the temperature increased from 20.0 to 42.5 °C. We observed that the aggregate diameter reached a maximum at a temperature around LCST. A further increase in temperature induced slight decrease in the diameter of the aggregates (42.0–48.0 °C) presumably because further heating the solutions triggered shrinking of the aggregates as a result of dehydration.^33,34^

Transmission electron microscopy was carried out to observe the actual morphologies of HIPEC-3 aggregates before and after heating to 37.3 °C as compared to those of the aggregates observed from DLS experiments. TEM images of HIPEC-3 aggregates are shown in Fig. 3. Before heating, aggregates with an average diameter of about 90 nm were visible (Fig. 3(b)). After heating to 38 °C, large aggregates were observed (Fig. 3(c)). It is worth noting that the aggregate sizes below LCST observed by TEM were smaller than those when measured by DLS. The probable reason for this result is that water evaporation caused aggregate dehydration and then resulted in the collapse and shrinkage of the aggregates in the TEM experiment.^35^

### 3.4. *In vitro* drug release behaviors of AmpB-HIPEC-aggregates

**Fig. 4:**
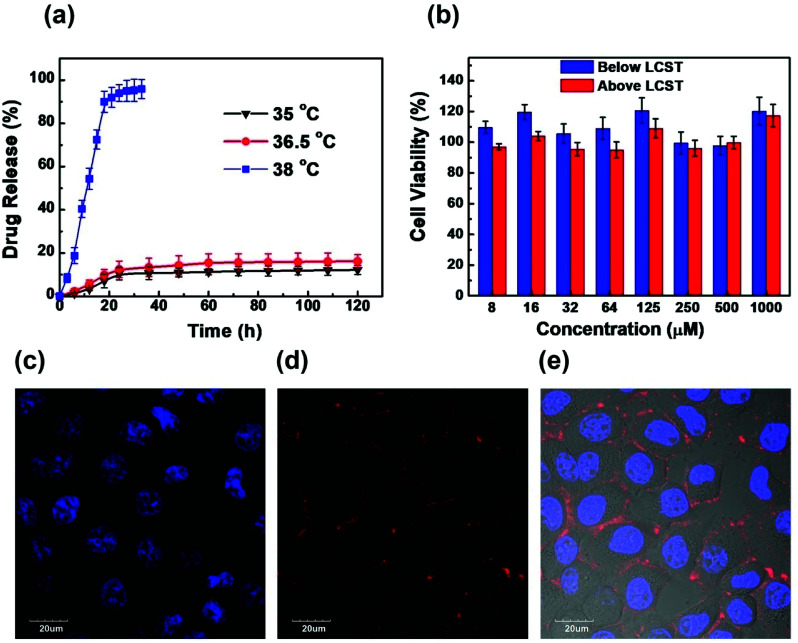
(a) Drug release profiles of AmpB-loaded HIPEC-3 aggregates at 35 °C, 36.5 °C and 38 °C in water. (b) Cytotoxicity of HIPEC-3 on normal hepatocytes (HL-7702) at temperatures below and above the LCST. (c, d) Confocal fluorescence images of HL-7702 cells incubated by PI and Hoechst 33342 at the same time. (e) Merged image of (c) and (d), and the corresponding bright-field image. (Blue color: Hoechst 33342 staining occurring in the cell nucleus, *λ*_ex_ = 405 nm, *λ*_em_ = 421–501 nm; red color: PI background fluorescence occurring outside the cells, *λ*_ex_ = 559 nm, *λ*_em_ = 577–657 nm).

Lipophilic drugs can be encapsulated and stabilized in the hydrophobic core of amphiphilic HIPEC aggregates. Amphotericin B, a lipophilic drug for the treatment of systemic mycosis, was used as a model drug (loading efficiency of 35.5%) to investigate the temperature-dependence release behaviors of HIPEC-aggregates.^28,36^ The *in vitro* release experiment of AmpB was conducted at 35.0 °C, 36.5 °C (below LCST) and 38 °C (above LCST) using AmpB-loaded HIPEC-3. As shown in Fig. 4(a), the release speed of AmpB was relatively slow at 35.0 °C and 36.5 °C. After 120 h, only 12% of the drug was released from the HIPEC-3 aggregates. In contrast, the release rate of AmpB was much faster at 38 °C. Specifically, nearly 95% of the drug was released from the aggregates in 40 h. Above the LCST, the hydrophilic shell collapsed, leading to a deformed aggregate structure. This, in turn, exposed the AmpB molecules to the solutions.

### 3.5. Cytocompatibility

The cytotoxicity of HIPEC-3 aggregates was investigated by normal hepatocytes (HL-7702) using MTT assays and confocal laser scanning microscopy (CLSM). As shown in Fig. 4(b), the cells presented very high viability even at high concentrations of HIPEC-3. The cell survival rates were above 95% at HIPEC-3 concentrations ranging from 8 to 1000 μM, indicating the excellent biocompatibilities of the HIPEC-3 aggregates in HL-7702 cells. In addition, we confirmed the cytotoxicity results of the MTT assays by confocal fluorescence microscopy. The cells were stained with propidium iodide (PI) and Hoechst 33342 at the same time. PI is membrane-impermeant, generally excluded from viable cells, and is commonly used for identifying dead cells in a population (nuclear staining). On the contrary, Hoechst 33342, which is a cell permeable nucleic acid stain and supravital minor groove-binding DNA stain with AT selectivity, is used to stain living cells (nuclear staining). As illustrated in Fig. 3(c–e), PI did not enter the cells, and Hoechst 33342 stained the cell nucleus well. Because of the charge attraction between the positively charged PI and the negatively charged cell membrane, PI, which cannot enter the viable cells, was attracted by the cell membrane rather than floating in the culture medium. The cell population showed high cell activity for HIPEC-3.

## 4. Conclusions

Thermoresponsive polymers with different degrees of substitution, *i.e.*, HIPECs were successfully synthesized *via* an etherification reaction and were characterized by ^1^H NMR, ^13^C NMR, and 2D HSQC NMR. The resulting HIPECs presented good thermoresponsivity with easily tunable LCST. LCSTs of the HIPECs linearly increased from 21.1 to 56.1 °C upon increasing the MS of hydrophobic groups. Meanwhile, the LCST values of the polymers were not apparently affected by slight variation in ion strength. Self-assembling aggregates of nano sizes were formed by dissolving HIPECs in aqueous solutions, and the aggregate diameter significantly increased when the temperature was above the LCST. As drug carriers, 95% of the drug was released from aggregates in 40 h when the temperatures were above the LCST. In addition, MTT assays and Hoechst 33342 and propidium iodide staining observed by confocal laser scanning microscopy confirmed the good biocompatibility of HIPECs. On the basis of the research results mentioned above, HIPECs are promising candidates for applications in targeted delivery of drugs.

## Conflicts of interest

There are no conflicts to declare.

## Supplementary Material

RA-009-C8RA09075K-s001
