# Supplementary material for: Thermoresponsive 2-hydroxy-3-isopropoxypropyl hydroxyethyl cellulose with tunable LCST for drug delivery
Source: RSC Adv. 2019 Jan 16;9(4):2268–76. doi: 10.1039/c8ra09075k (PMC9059852; doi:10.1039/c8ra09075k)
Supplement: RA-009-C8RA09075K-s001 [file RA-009-C8RA09075K-s001.pdf]

Electronic Supplementary Material (ESI) for RSC Advances.
This journal is © The Royal Society of Chemistry 2019

Electronic Supplementary Information

Thermoresponsive 2-Hydroxy-3-Isopropoxypropyl Hydroxyethyl Cellulose with Tunable LCST for Drug Release

Ye Tian, *[a,c] Benzhi Ju[b], Ying Liu,[a] Xiaozhong Ren[a], Mingyun Dai[a]

a. Dalian Ocean University, Aquacultural Engineering Research and Development Center, Dalian 116023, China.
b. State Key Laboratory of Fine Chemicals, Dalian University of Technology, Dalian 116023, China.
c. Key Laboratory of Mariculture & Stock Enhancement in North China's Sea, Dalian 116023, China.

Correspondence to

E-mail: tianye@dlou.edu.cn (Y. Tian); jubenzhi@dlut. edu.cn (B. Ju)

Supplementary data__GPC Experimental

The molecular weights and molecular weight distribution of HIPEC were measured on an Agilent Technologies 1200 series gel permeation chromatograph equipped with two columns (ultrahydrogel 1000 7.8 mm×300 mm and ultrahydrogel 250 7.8 mm × 300 mm). Sample was dissolved in 1 mL of eluent (concentration 0.1%, w/w). Injection volume was 100 μL. H_2O was used as the eluent at a flow rate of 1 mL/min at 20 °C.

Supplementary data__^{1}H-NMR，^{13}C-NMR, and 2D HSQC NMR spectra

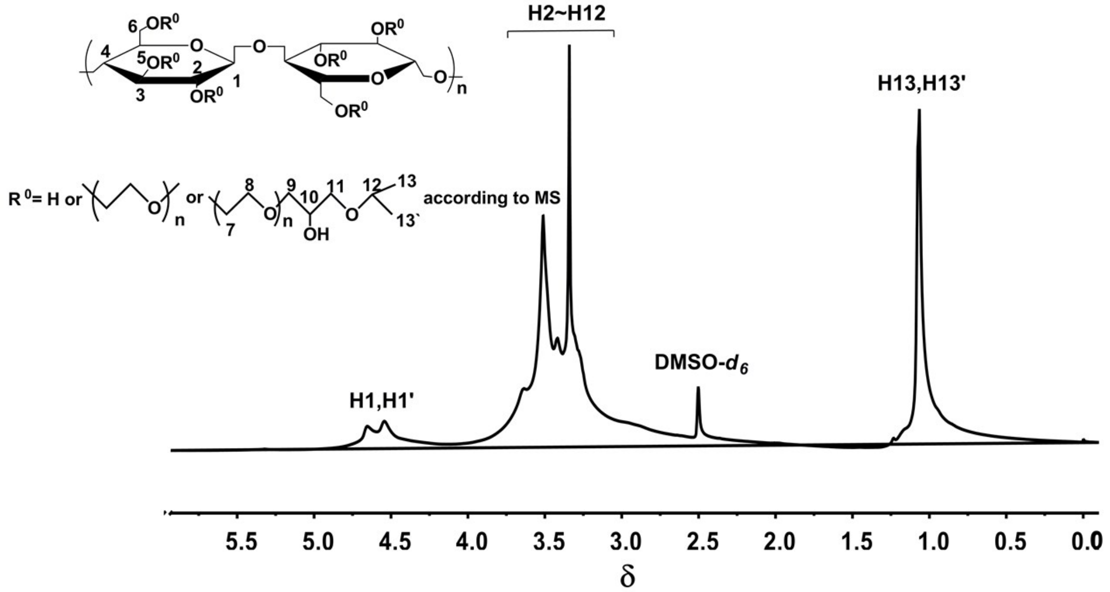

Figure S1. ^{1}H-NMR spectra of HIPEC-3 recorded in DMSO-d_6

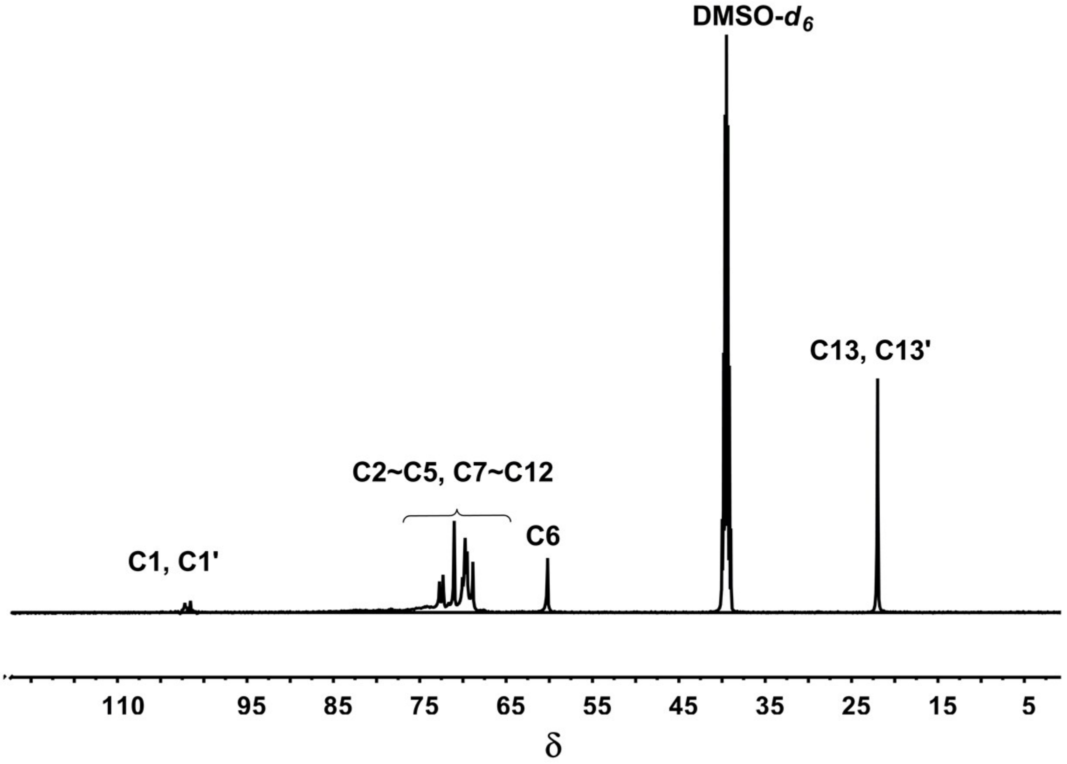

Figure S2. ^{13}C-NMR spectra of HIPEC-3 recorded in DMSO-d_6

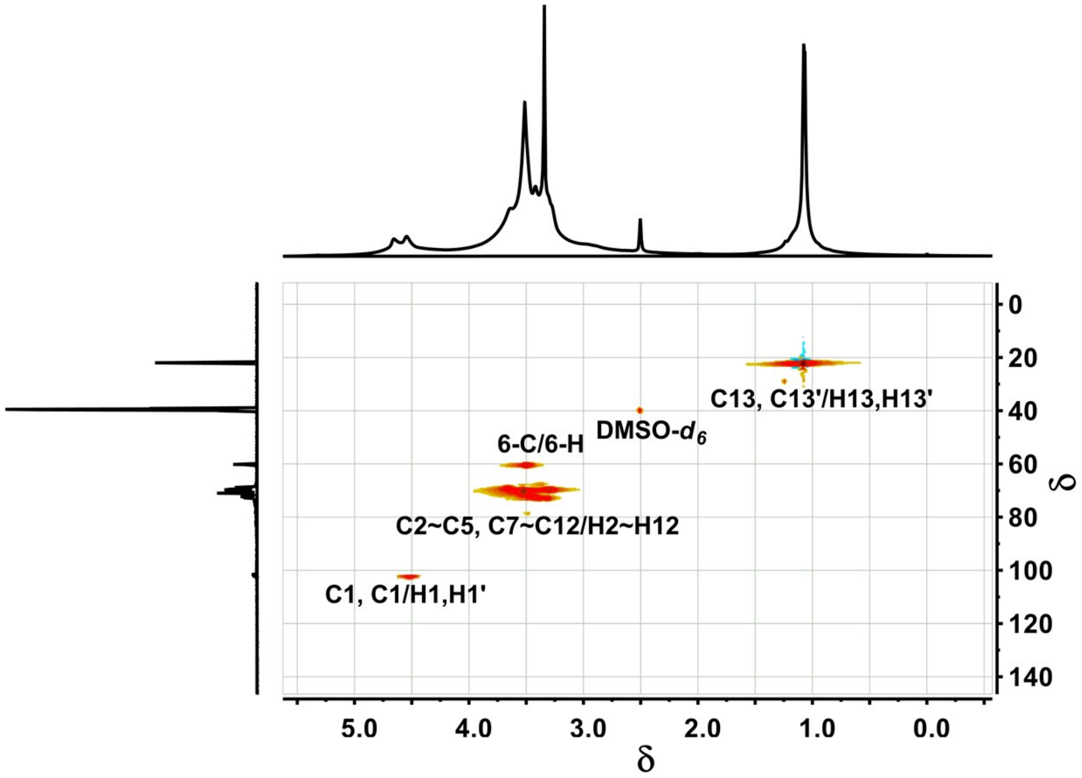

Figure S3. 2D HSQC NMR spectra of HIPEC-3 recorded in DMSO-d_6
